# Thinking inside the box: intracellular roles for complement system proteins come into focus

**DOI:** 10.1038/s41416-022-02116-7

**Published:** 2023-01-17

**Authors:** Rebecca M. O’Brien, Niamh Lynam-Lennon, Monica M. Olcina

**Affiliations:** 1 Department of Surgery, Trinity St. James’s Cancer Institute, Trinity Translational Medicine Institute, Trinity College Dublin, St. James’s Hospital, Dublin 8, Ireland; 2 Cancer Immunology and Immunotherapy Group, Trinity St. James’s Cancer Institute, Trinity Translational Medicine Institute, Trinity College Dublin, St. James’s Hospital, Dublin 8, Ireland; 3 Medical Research Council Oxford Institute for Radiation Oncology, Department of Oncology, University of Oxford, Old Road Campus Research Building, Off Roosevelt Drive, Oxford, OX3 7DQ UK

**Keywords:** Cancer microenvironment, Oncology

## Abstract

Over the last decade, perspectives on the complement system in the context of cancer have shifted, with complement proteins now implicated in many of the hallmarks of cancer. Systemically, the generation of complement anaphylatoxin C5a, the most potent inflammatory mediator of the cascade, occurs following convertase-mediated cleavage of complement component C5. In a recent manuscript, Ding et al., propose that in colorectal cancer cells, C5 cleavage can occur intracellularly and in a convertase-independent manner, identifying cathepsin D as an enzyme capable of cleaving C5 into C5a [1]. Intracellular C5a is functional and promotes β-catenin stabilisation via the assembly of a KCTD5/cullin3/Roc-1 complex. Importantly, the blockade of C5aR1 prevents tumorigenesis. This study adds to a growing body of evidence indicating that complement proteins, previously thought to primarily have extracellular or membrane-bound functions, also have important intracellular roles.

Historically, the complement system has been regarded as a group of circulating, liver-derived proteins, important for pathogen opsonisation, lysis and danger sensing [2]. More recently, increasing evidence demonstrates that complement components are also produced within the tumour microenvironment (TME) by both stromal and cancer cells. A seminal paper by Markiewski et al. in 2008 [3] first elucidated the importance of complement in promoting tumour growth. This study showed that TME-generated C5a, (acting on host receptors), suppresses anti-tumour CD8^+^ T-cell responses via regulation of reactive oxygen and nitrogen species in myeloid-derived suppressor cells (MDSCs). Using a syngeneic mouse model of cervical cancer, they demonstrated that C5a receptor (C5aR) antagonism or C5aR1 genetic deficiency controlled tumour growth to a similar extent to paclitaxel [3]. Subsequent studies have confirmed the importance of complement proteins for the response to chemotherapy, radiotherapy and immunotherapy (reviewed in ref. [4]). Supporting a role for complement proteins in cancer, dysregulation of members of this pathway (through either altered mRNA, protein expression or genetic alterations) is associated with changes in survival outcome [1, 5]. As the roles of complement within the TME have come into focus, intracellular complement has also emerged as an important regulator of oncogenesis. In their recent publication, Ding et al. [1] provide evidence for an intracellular C5a/C5aR1 signalling axis in colorectal cancer (CRC) cells (Fig. 1).

**Fig. 1:**
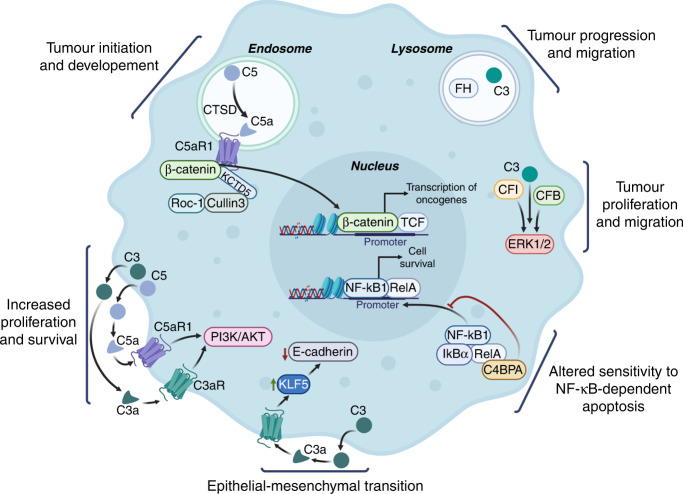
Schematic representation of recently described roles for intracellular complement proteins in cancer cells. Figure created in BioRender. Agreement number: IQ24HOC297.

Systemically, the complement effectors C3a and C5a are typically generated via convertase-mediated cleavage of central complement system proteins C3 and C5. However, Ding et al. propose that in CRC cells, C5 cleavage occurs in a convertase-independent manner, identifying cathepsin D as an enzyme capable of cleaving C5 into C5a [1]. These data suggest that in cancer, intracellular complement activation yields anaphylatoxins which potentially initiate downstream signalling. Addressing this possibility, Ding et al. observe a relationship between intracellular C5a/C5aR1 and expression levels of β-catenin, a known promoter of oncogene transcription and contributor to CRC carcinogenesis [1]. In HCT-15 colonic cancer cells, C5 or C5aR1 deficiency results in decreased protein expression of β-catenin [1]. Similarly, reduced β-catenin protein levels are observed following treatment with a C5aR1 antagonist (at high concentrations) in human and murine colonic cell lines. These experiments suggest that C5a/C5aR signalling stabilises β-catenin expression [1]. Using immunoprecipitation and mass spectrometry, Ding et al. show that C5aR1 interacts with a KCTD5/cullin3/Roc-1 complex. Assembly of this complex is triggered by C5a, and favours β-catenin stabilisation [1]. These experiments demonstrate that intracellular complement signalling can potentially facilitate the acquisition of an oncogenic phenotype. Importantly, in a chronic colitis-induced murine model of CRC, C5aR1 blockade prevents tumorigenesis. This is associated with reduced protein expression of β-catenin, providing further evidence that C5a/C5aR1 signalling promotes β-catenin stabilisation [1]. Interestingly, in support of in vitro and in vivo findings, β-catenin levels and C5a/C5aR1 levels are strongly correlated in clinical samples from patients with colorectal adenomas and adenocarcinomas [1]. This study ultimately highlights how intracellular complement may provide insights into the active pathways driving tumorigenesis.

This recent work by Ding et al. adds to a growing body of evidence indicating that complement proteins, previously thought to predominantly have extracellular or membrane-bound functions, also have important intracellular roles. Liszewski et al. showed that cathepsin L-mediated cleavage of C3 generates C3a and C3b in T cells. C3a was demonstrated to play an essential role in homoeostasis in naïve CD4^+^ T cells, while intracellular C5a is now known to orchestrate the induction of a T helper 1 phenotype [6, 7]. Recent work has also highlighted that an intracellular mitochondrial C5a/C5aR1 signalling axis modulates sterile inflammation in myeloid cells [8]. In this context, the assembly of an intracellular C5 convertase was proposed to cleave C5 to produce C5a, which engages C5aR1 and promotes IL-1B production [8]. Together, these studies demonstrated that intracellular activation of complement and subsequent anaphylatoxin signalling can alter key cellular phenotypes in immune cells. Whether these intrinsic complement signalling axes regulate immune populations in the TME remains to be completely determined.

Furthermore, whether convertases remain intact in the reducing conditions present in the cytoplasm has been a contentious issue, and whether convertases are stable and active inside cancer cells is largely unexplored. However, in cancer, complement proteins have been found in the oxidising environment of endosomes and lysosomes, where these disulfide-rich proteins may be more stable [1, 9, 10]. In clear cell renal cell carcinoma and lung adenocarcinoma, complement factor H (FH) has been identified in lysosomes where it engages in a pro-tumour role distinct from its membranous counterpart [10]. Independent of the complement system, FH promotes tumour cell proliferation, migration and survival and is associated with poor patient outcomes [10]. Given the highly genomically unstable nature of cancers, it will be relevant to further investigate if the subcellular localisation of intracellular complement proteins changes in response to the mutational background and the selective pressures of cancer treatment. In CRC cells harbouring mutations in C4b-binding protein alpha chain (C4BPA), this complement-associated protein is retained in the cytoplasm in response to the chemotherapy agent oxaliplatin [11]. Increased intracellular C4BPA expression in this context is relevant since C4BPA interacts with RelA, a member of the NF-κB family, to enhance apoptosis following oxaliplatin treatment [11]. As in the study from Ding et al., these studies demonstrate that once expressed intracellularly, complement proteins can have roles independent from their ‘canonical’ complement system functions (Fig. 1).

An emerging theme is that complement proteins are often hijacked by tumour cells for their survival advantage, and this clearly includes complement proteins present intracellularly. Cancer cells appear reliant on these hijacked proteins for survival which suggests that therapeutically targeting these intracellular complement components could maximise therapeutic responses. For successful therapeutic targeting, it will be key to establish the structural properties and forms of intracellular complement proteins. For example, central complement component C3 can be translated from an alternative start site, resulting in a protein lacking its signal peptide, which is expressed primarily in the cytosol in a non-glycosylated reduced form [12]. Whether this form of C3 is present widely across cancer cells is yet to be determined. To what extent the re-uptake of complement proteins from the extracellular space contributes to intracellular pools also remains to be comprehensively assessed.

Importantly, these new findings highlight that further study of intracellular complement has the potential to elucidate molecular mechanisms underlying therapeutic resistance and reveal novel therapeutic targets. Considering the recent finding that C5aR1 signalling stabilises β-catenin, which is a protein associated with poor patient prognosis, we postulate that establishing the interactome of intracellular complement components may also identify new prognostic and predictive biomarkers [1].

## References

[1] Ding P, Xu Y, Li L, Lv X, Li L, Chen J (2022). Intracellular complement C5a/C5aR1 stabilizes b-catenin to promote colorectal tumorigenesis. Cell Rep.

[2] Ricklin D, Hajishengallis G, Yang K, Lambris JD (2010). Complement: A key system for immune surveillance and homeostasis. Nat Immunol.

[3] Markiewski MM, Deangelis RA, Benencia F, Ricklin-Lichtsteiner SK, Koutoulaki A, Gerard C (2008). Modulation of the anti-tumor immune response by complement. Nat Immunol.

[4] O’Brien RM, Cannon A, Reynolds JV, Lysaght J, Lynam‐Lennon N (2021). Complement in tumourigenesis and the response to cancer therapy. Cancers (Basel).

[5] Olcina MM, Balanis NG, Kim RK, Aksoy BA, Kodysh J, Thompson MJ (2018). Mutations in an innate immunity pathway are associated with poor overall survival outcomes and hypoxic signaling in cancer. Cell Rep.

[6] Liszewski MK, Kolev M, Le Friec G, Leung M, Bertram PG, Fara AF (2013). Intracellular complement activation sustains T cell homeostasis and mediates effector differentiation. Immunity.

[7] Arbore G, West EE, Spolski R, Robertson AAB, Klos A, Rheinheimer C (2016). T helper 1 immunity requires complement-driven NLRP3 inflammasome actvity in CD4+ T cells. Science.

[8] Niyonzima N, Rahman J, Kunz N, West EE, Freiwald T, Desai JV, et al. Mitochondrial C5aR1 activity in macrophages controls IL-1β production underlying sterile inflammation. Sci Immunol. 2022. 10.1126/sciimmunol.abf2489.Mitochondrial. 10.1126/sciimmunol.abf2489 PMC8902698 34932384

[9] Austin CD, Wen X, Gazzard L, Nelson C, Scheller RH, Scales SJ (2005). Oxidizing potential of endosomes and lysosomes limits intracellular cleavage of disulfide-based antibody-drug conjugates. Proc Natl Acad Sci USA.

[10] Daugan MV, Revel M, Thouenon R, Dragon-Durey MA, Robe-Rybkine T, Torset C (2021). Intracellular factor H drives tumor progression independently of the complement cascade. Cancer Immunol Res.

[11] Olcina MM, Kim RK, Balanis NG, Li CG, von Eyben R, Graeber TG (2020). Intracellular C4BPA levels regulate NF-κB dependent apoptosis. iScience.

[12] Kremlitzka M, Colineau L, Nowacka AA, Mohlin FC, Wozniak K, Blom AM, et al. Alternative translation and retrotranslocation of cytosolic C3 that detects cytoinvasive bacteria. Cell Mol Life Sci. 2022. 10.1007/s00018-022-04308-z. 10.1007/s00018-022-04308-z PMC9095555 35546365

